# Extravascular Lung Water Correlates Multiorgan Dysfunction Syndrome and Mortality in Sepsis

**DOI:** 10.1371/journal.pone.0015265

**Published:** 2010-12-16

**Authors:** Fu-Tsai Chung, Horng-Chyuan Lin, Chih-Hsi Kuo, Chih-Teng Yu, Chun-Liang Chou, Kang-Yun Lee, Han-Pin Kuo, Shu-Min Lin

**Affiliations:** 1 Department of Thoracic Medicine, Chang Gung Memorial Hospital at Linkou, Chang Gung University, College of Medicine, Taipei, Taiwan, Republic of China; 2 Graduate Institute of Clinical Medical Sciences, College of Medicine, Chang Gung University, Taipei, Taiwan, Republic of China; Fundação Oswaldo Cruz, Brazil

## Abstract

**Background:**

This study was designated to investigate whether increased extravascular lung water index (EVLWI) may correlate multiple organ dysfunction syndrome (MODS) and mortality in sepsis.

**Methods:**

We designed a prospective cohort study in an intensive care unit of a tertiary care hospital. Sixty-seven patients with severe sepsis were included. Data were used to determine an association between EVLWI and the development of MODS and mortality. These connections were determined by the multiple logistic regression, plotting the receiver operating characteristic (ROC) curve and by Spearman test.

**Results:**

EVLWI levels were higher in MODS patients on day 1 (median (IQR), 18(12.8–23.9) ml/kg, n = 38, *p*<0.0001) than in those without (median (IQR), 12.4 (7.9–16.3) ml/kg, n = 29) and day 3 (median (IQR), 17.8 (11.2–22.8) ml/kg, n = 29, *p* = 0.004) than in those without (median (IQR), 12.4 (8.0–16.3) ml/kg, n = 29). EVLWI was used as an independent predictor of the development of MODS (odds ratio, 1.6; *p* = 0.005; 95% confidence interval, 1.2∼2.2) during ICU stay. The area under the ROC curve showed that EVLWI levels could predict MODS (0.866) and mortality (0.881) during ICU stay. Meanwhile, the higher of SOFA score, the more EVLWI was found on day 1 (r = 0.7041, p<0.0001) and day 3 (r = 0.7732, p<0.0001).

**Conclusions:**

Increased EVLWI levels correlates development of MODS and mortality during the patients' ICU stay. Further more, the potential of novel treatment in severe sepsis with lung injury may develop.

## Introduction

The incidence of sepsis has been increasing because of the advancing age of the general population, a greater number of invasive procedures, and more immunosuppressive therapies [1]. Despite antimicrobial agents and advanced life-support care, the fatality rate for sepsis remains at 30–40% [1]. Severe sepsis is frequently complicated by multiple organ dysfunction syndrome (MODS). MODS means two or more organs are dysfunctional; however, when three or more organs are involved, MODS cause 60% to 98% death of severe sepsis [2], [3]. Over 30 years after its first report, the mortality of MODS is still high, and is still the major cause of morbidity and mortality for sepsis patients admitted to an intensive care unit, and the costs of treatment are huge [4], [5]. Meanwhile, sepsis-induced MODS frequently leads to death in patients with sepsis [6], [7]. Certainly, the severity of organ dysfunction is an important determinant of prognosis in sepsis [8], [9]. However, despite MODS is important in the patients with severe sepsis, the pathogenesis including endothelial injury and permeability change in sepsis and MODS is not clear.

Endothelial activation and damage occur early during sepsis [10]. The activation and damage of endothelial cells are closely related to organ dysfunction because these cells cover the surfaces of blood vessels and are in close contact with organs [10]. During sepsis, exposure to inflammatory mediators and interaction with leukocytes causes endothelial activation and damage [11]. The sepsis-induced damage of endothelial cell membranes gives rise to increased capillary permeability [12], [13]. The increased systemic vascular permeability may occur within hours of an acute event such as surgery or sepsis, causing an extravasation of albumin and water leading to interstitial edema [14]. The hyperpermeability state plays an important role in mediating tissue ischemia and organ failure in sepsis [15]. Evidence suggests that increased capillary permeability during the first 48 h in patients with sepsis was associated with a higher mortality rate during the intensive care unit (ICU) stay than those with decreased permeability [16], [17].

The increased capillary permeability manifests in the lungs as altered alveolar–capillary barrier function and is characterized by accumulation of extravascular lung water index (EVLWI). Among the methods measuring EVLWI, the thermal-dye dilution method is considered to be the “gold standard” of EVLWI measurements *in vivo*. EVLWI determined with the single transpulmonary thermodilution technique correlates with that determined with the thermal-dye dilution [18], [19] and the new technique has been well validated in critically ill patients [20]. Previous work has reported the prognostic value of extravascular lung water (EVLW) in patients with acute lung injury [21]. Our recent work demonstrated that severe sepsis patients with elevated EVLWI were more vulnerable to mortality during their hospital stay [22]. However, few comprehensive studies have investigated the relationship between EVLW and the development of MODS in patients with severe sepsis.

This study was designed to investigate whether EVLWI is an independent factor for MODS in patients with severe sepsis.

## Results

A total of 67 patients were enrolled in this study. There were 38 (56.7%) patients with MODS on day 1. In the cohort, 56 patients required mechanical ventilation. The characters of patients with and without MODS on day 1 and 3 were listed in table 1. In table 1, the fluid balance was significantly different on day 1 but not on day 3. However, EVLWI remained higher in patients with MODS on day1 and 3. The day 3 EVLWI and fluid balance support that fluid balance is not the main reason to cause high EVLWI in patients with MODS. In addition, the pulmonary permeability index (PPI) of patients with MODS was higher than those without MODS on day1 and day 3.

**Table 1:** Characteristics of patients with and without MODS at days 1 and 3 of severe sepsis.

		Day 1			Day 3	
Variables	MODS	Without MODS	*p* value	MODS	Without MODS	*p* value
	n = 38	n = 29		n = 29	n = 29	
Age (years)	70 (50–80)	70 (54–77)	NS	68 (54–77)	70 (54–77)	NS
Male gender	31 (81.6)	19 (65.5)	NS	23 (74.2)	19 (65.5)	NS
APACHE II score	27 (24–32)	21 (18–26)	0.0008	24 (20–31)	19 (15–22)	0.01
Body mass index (kg/m^2^)	19.9 (18.2–24.9)	22.5 (18.9–27.4)	0.01	20.1 (18.5–23.8)	22.5 (18.9–27.4)	NS
Prior 24 hrs fluid balance (L)	3.1 (1.9–4.0)	2.5 (1.1–3.2)	0.0008	1.8 (1.3–3.0)	1.6 (1.1–2.0)	NS
PPI	3.8 (2.7–6.3)	1.8 (1.5–2.1)	0.0005	2.9 (2.1–4.0)	1.7 (1.3–2.3)	0.01
EVLWI (mL/kg)	18 (12.8–23.9)	12.4 (7.9–16.3)	<0.0001	17.8 (11.2–22.8)	12.4 (8.0–16.3)	0.004
PaO_2_/FiO_2_ ratio	149 (75–217)	190 (102–268)	0.02	186 (128–239)	211 (162–259)	NS
CXR score	3.0 (2.0–3.0)	2.0 (1.0–3.0)	NS	3.0 (2.0–3.0)	2.0 (1.0–2.0)	NS
Lung injury score	2.6 (2.0–3.3)	1.6 (1.3–2.0)	0.006	2.4 (1.6–3.0)	1.3 (1.0–2.0)	0.02
Albumin (g/L)	0.019 (0.016–0.023)	0.023 (0.017–0.024)	NS	0.021 (0.018–0.022)	0.022 (0.018–0.025)	NS
Platelet (10^9^/L)	101 (78.3–174)	146 (74–224)	NS	121 (75–157)	178 (102–245)	NS
WBC (10^9^/L)	17.7 (13.1–21.9)	16.7 (12.1–21.9)	NS	15.4 (12.2–17.8)	15.6 (11.2–16.5)	NS
SOFA score	11 (10–13)	6 (4–7)	0.0001	10 (7–11)	4 (2–7)	0.0001
PEEP (cm cmH_2_O)	10 (8–12)	8 (8–10)	0.01	8 (6–10)	6 (6–10)	0.04
Compliance of lung (mL/cmH_2_O)	46 (37–54)	69 (59–79)	0.001	53 (40–69)	66 (55–79)	0.03
Plateau pressure (cmH_2_O)	23 (21–25)	20 (16–22)	0.001	21 (19–24)	19 (17–22)	0.03
Mean airway pressure	15 (14–16.7)	14 (13–15)	0.01	15 (14–16)	14 (13–15)	NS
Tidal volume (mL/kg)	5 (5–6)	7 (6–8)	0.001	6 (5–6)	7 (6–8)	0.02
Minute ventilation (L/min)	10.8 (10.4–12.5)	10.2 (9.1–10.9)	0.01	10.8 (9.2–12.1)	10.5 (9.2–10.8)	NS
Vasopressor use	36 (94.7)	21 (72.4)	0.01	17 (54.8)	9 (33.3)	NS

*Definitions of abbreviation*: MODS =  multi-organs dysfunction syndrome; APACHE =  acute physiology and chronic health evaluation; PPI, pulmonary permeability index; EVLWI =  extravascular lung water index; PaO2 =  partial pressure of oxygen in arterial blood; FiO2 =  fraction of inspired oxygen; CXR =  chest X-ray; WBC =  white blood cell; SOFA =  Sequential Organ Failure Assessment score; PEEP =  positive end-expiratory pressure.

Values are expressed as median (interquartile range) or numbers (%).

NS =  Non-significant, *p*>0.05.

Univariate analyses were primarily used for the selection of variables, based on a *p* value less than 0.05 (Table 1). The selected variables including SOFA score, APACHE II score, BMI, fluid balance 24 hrs prior, vasopressore use, PPI, EVLWI, PaO2/FiO2 ratio, lung injury score, PEEP and compliance of the lung were further analyzed by multiple logistic regression analysis. The results are presented in Table 2. EVLWI (odds ratio, 1.6; *p* = 0.005; 95% confidence interval, 1.2∼2.2), SOFA score (odds ratio, 1.7; *p* = 0.03; 95% confidence interval, 1.0∼3.1) and APACHE II score (odds ratio, 1.1; *p* = 0.04; 95% confidence interval, 1.0–1.3) remained significant predictors of MODS development during ICU stay after controlling for other variables. BMI, prior 24 hrs fluid balance, vasopressore use, PaO2/FiO2 ratio, lung injury score, PEEP and compliance of the lung failed to maintain their prognostic value for the MODS development during ICU stay in the adjusted analysis.

**Table 2:** Multivariate analysis of major factors associated with multiple organs dysfunction syndrome in patients with severe sepsis.

Variables	Odds ratio	95% C.I.	*p* value
EVLWI*	1.6	1.2–2.2	0.005
SOFA score*	1.7	1.0–3.1	0.03
APACHE II score*	1.1	1.0–1.3	0.04
Lung injury score	2.5	0.2–9.2	0.48
Prior 24 hrs fluid balance	1.1	0.9–1.2	0.47
Body mass index	1.1	0.8–1.4	0.57
Vasopressor use	2.9	0.4–10.4	0.34
PaO_2_/FiO_2_ ratio	1.1	0.9–1.2	0.18
PEEP	1.6	0.5–5.6	0.46
Compliance of lung	0.9	0.8–1.1	0.37

*Definitions of abbreviation*: EVLWI =  extravascular lung water index; SOFA =  Sequential Organ Failure Assessment score; APACHE =  acute physiology and chronic health evaluation; PaO2 =  partial pressure of oxygen in arterial blood; FiO2 =  fraction of inspired oxygen; PEEP =  positive end-expiratory pressure.

*p value for difference between groups <0.05.

**Figure 1:**
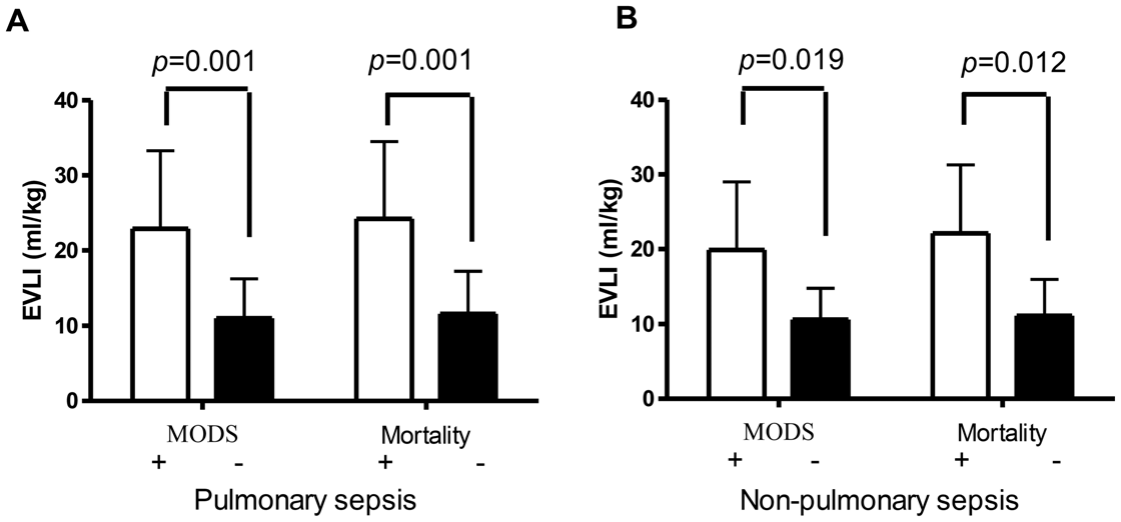
Extravascular lung water index (EVLWI). EVLWI in patients with and without multi-organ dysfunction syndrome (MODS), and mortality in pulmonary sepsis (A) and in non-pulmonary sepsis patients (B), *Open bars*, patients with MODS or mortality; *filled bars*, patients without MODS or mortality. *p* value was expressed.

Figure 1A shows day 1 EVLWI levels in pulmonary sepsis patients with and without MODS and in survivors and fatalities during ICU stay. EVLWIs were higher in MODS patients (23.1±11 ml/kg, n = 29, *p* = 0.001) than in those without (11.2±5.1 ml/kg, n = 17). Similarly, fatalities had increased levels of EVLWIs (25.0±10.7 ml/kg, n = 26) compared with survivors (11.8±5.9 ml/kg, n = 20, *p* = 0.001). In 21 patients with sepsis from non-pulmonary origin (blood stream infection = 9, urosepsis = 9, skin and subcutaneous infection = 3), EVLWIs were higher in MODS patients (21.8±10.4 ml/kg, n = 9, *p* = 0.019) than in those without (11.4±5.3 ml/kg, n = 12). Similarly, fatalities had increased levels of EVLWIs (22±10.3 ml/kg, n = 7) compared with survivors (12.8±5.7 ml/kg, n = 14, *p* = 0.012).

**Figure 2:**
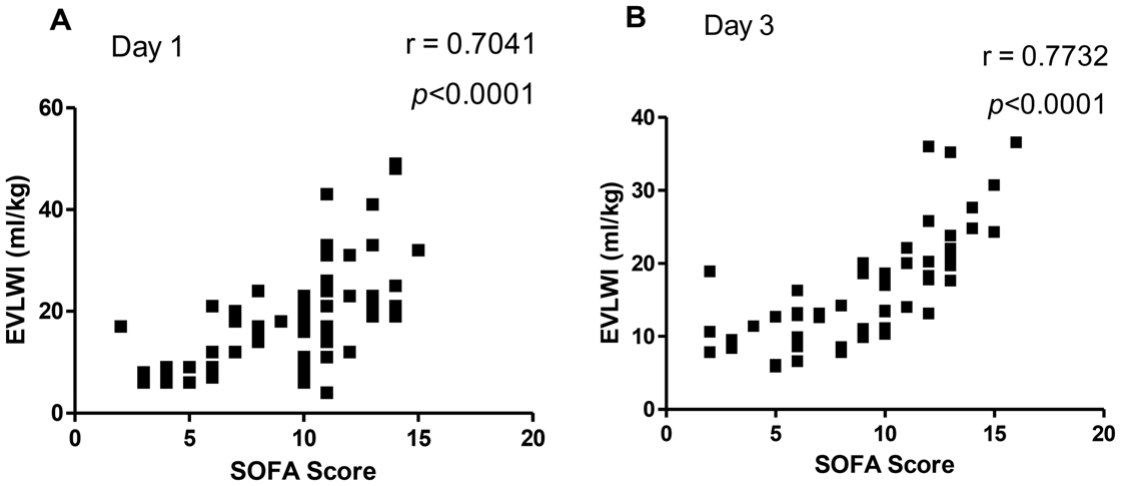
The relationship between extravascular lung water index levels (EVLWI) and SOFA score in patients with severe sepsis. The higher of SOFA score, the more EVLWI was found on day 1 (r = 0.7041, p<0.0001) and day 3 (r = 0.7732, p<0.0001). The data are expressed as Spearman test.

Figure 2 demonstrates the relationship between extravascular lung water index levels (EVLWI) and SOFA score in patients with severe sepsis. The higher of SOFA score, the more EVLWI was found on day 1 (r = 0.7041, p<0.0001) and day 3 (r = 0.7732, p<0.0001). The data are expressed as Spearman test.

Comparisons between areas under the ROC curve for EVLWI, SOFA score, APACHE II scores, lung injury score, and prior 24 hrs fluid balance on day 1 of severe sepsis in prediction of clinical outcomes during ICU stay are listed in Table 3. Values for areas under the ROC curves showed that day 1 EVLWI levels could be used to predict MODS (0.866) and mortality (0.881) during ICU stay. The areas under the ROC curves for day 1 SOFA score and APACHE II scores were similar in predicting ICU mortality (SOFA score, 0.730; APACHE II score, 0.818) as lung injury score (0.740), and prior 24 hrs fluid balance (0.725) but less than EVLWI. However, the areas under the ROC curves for day 1 SOFA score was adequate in predicting MODS (0.848) development during ICU stay rather than APACHE II scores (0.747), lung injury score (0.692), and prior 24 hrs fluid balance (0.729) but also less than EVLWI.

**Table 3:** Comparison among areas under the receiver operating characteristic (ROC) curves for variables on day 1 of severe sepsis, n = 67.

	Area Under ROC Curve	
	MODS (95% C.I.)	Mortality (95% C.I.)
EVLWI>10 ml/kg	0.866 (0.778–0.954)	0.881 (0.801–0.960)
SOFA score	0.848 (0.760–0.937)	0.730 (0.608–0.851)
APACHE II score	0.747 (0.628–0.866)	0.818 (0.713–0.923)
Lung injury score	0.692 (0.566–0.818)	0.740 (0.619–0.862)
Prior 24 hrs fluid balance	0.729 (0.608–0.850)	0.725 (0.604–0.846)

*Definitions of abbreviation*: MODS =  multiple organs dysfunction syndrome; C.I. =  confidence interval; EVLWI =  extravascular lung water index; SOFA =  Sequential Organ Failure Assessment score; APACHE =  acute physiology and chronic health evaluation.

Sensitivities, specificities, and predictive values of elevated EVLWI (≥10 ml/kg) for the development of MODS and mortality during ICU stay are shown in table 4. The sensitivity of elevated EVLWI was 85% in MODS and 94.7% in ICU mortality. The specificity of increased EVLWI was 75% in MODS and 66.7% in ICU mortality. The positive and negative predictive value of increased EVLWI was 58.6% and 92.3% in MODS and 52.9% and 97% in ICU mortality, respectively.

**Table 4:** Diagnostic sensitivity, specificity, and predictive value of EVLWI >10 ml/kg for multiple organs dysfunction syndrome and mortality during ICU stay.

	MODS, %	Mortality, %
Sensitivity	85.0	94.7
Specificity	75.0	66.7
Positive predictive value	58.6	52.9
Negative predictive value	92.3	97.0

*Definitions of abbreviation*: EVLWI =  extravascular lung water index; ICU =  intensive care unit; MODS =  multiple organs dysfunction syndrome.

## Discussion

This study has demonstrated that EVLWI and APACHE II scores are independent factors for the development of MODS in patients with severe sepsis. Increased EVLWI was associated with MODS on the day of the patients' ICU admission and subsequent development of organ failure during their ICU stay. With the cut-off value of 10 ml/kg, EVLWI offered good diagnostic sensitivity, specificity, and negative predictive value for MODS and mortality in those patients.

The APACHE II score and SOFA score have already been used to evaluate the illness severity of ICU patients [25], [27]. The increased APACHE II scores and SOFA score in septic patients may indicate higher illness severity, and is therefore associated with the development of MODS [25], [27]. Previous studies have suggested that EVLWI may serve as a prognostic marker for patients with sepsis [20], [22], [28]. Bognar and associates observed EVLWI elevation one day before the clinical manifestation of sepsis in burned patients [29]. Thus EVLWI may be recognized as an early sign of developing sepsis in burns. The sepsis-induced damage to the pulmonary microvasculature primarily increases the permeability of the endothelial membrane to fluids, hence resulting in capillary leakage and accumulation of extravascular lung water. The increased EVLWI, in turn, may result in arterial hypoxemia. The association between increased EVLWI and MODS may be explained by the severe hypoxemia. Our study showed that patients with MODS had elevated EVLWI and lower PaO2/FiO2 ratios than those without MODS on day 1 of severe sepsis. Similar trend on day 3 was noted, but the data was not statistical significant may be due to the number size decrease at day 3 with 9 patients died. This finding is in agreement with other study [21]. In addition, increased EVLWI indicates increased pulmonary permeability; the inflammatory mediators may leak into the systemic circulation [30]. The process can initiate or propagate a systemic inflammatory response and thus could play a role in the development of MODS. In our study, more patients with MODS developed acute lung injury and had lower lung compliance than patients without MODS. Therefore, these patients had lower tidal volume than those without MODS. The lower BMI in patients with MODS than those without MODS may be due to the presence of malnutrition since the albumin levels were also decreased in patients with MODS than those without MODS.

A recent study reported that 27% of severe sepsis patients who fulfilled the clinical consensus conference criteria for ARDS had never displayed raised EVLWI. In contrast, 57% of severe sepsis patients without clinical ARDS have increased EVLWI [28]. Therefore, the increased EVLWI in patients with sepsis could not be completely explained by lung injury. In fact, sepsis may cause increased EVLWI without causing ARDS. On table 1, the fluid balance was significantly different on day 1 but not on day 3. The reason may be due to the condition of patients with MODS more improved and less fluid needed than patients without MODS. However, EVLWI remained higher in patients with MODS on day 3. The day 3 EVLWI and fluid balance support that fluid balance is not the main reason to cause high EVLWI in patients with MODS. The results of the study also demonstrated that the permeability of patients with MODS was higher than those without MODS on day1 and day 3. Our results revealed that EVLWI was increased in patients with sepsis-induced MODS more than in those without. In a subgroup analysis, the EVLWI was also increased in patients with MODS and sepsis from both pulmonary and non-pulmonary origin (figure 1). Therefore, the sepsis of non-pulmonary origin may cause the increased pulmonary permeability and EVLWI. The increased EVLWI in patients with MODS was not completely caused by pneumonia-induced injury. Sepsis-induced increased pulmonary permeability also plays an important role in increased EVLWI in patients with sepsis-induced MODS.

With a cut-off value of 10, this study has demonstrated that patients with EVLWI >10 ml/kg on day 1 had a higher chance of MODS on day 1 of ICU admission and subsequent development of MODS during their ICU stay. Patients with increased day 1 EVLWI had higher chances of subsequent dysfunction of each organ system and are more prone to ICU mortality when compared with those without. The area under the ROC curve confirms that day 1 EVLWI levels are superior to APACHE II and lung injury score in predicting the development of MODS and mortality during patients' ICU stay. In addition, day 1 EVLWI offered good sensitivity, specificity, and negative predictive values for MODS and ICU mortality.

There are some limitations in current study. First, Frederic Michard reported the limitations of dilution methods may lead to an underestimation of EVLW in large pulmonary vascular obstruction, focal lung injury, and lung resection, but dilution methods remains an easy and clinically acceptable estimation of EVLW in most critically ill patients, including those with acute respiratory distress syndrome (ARDS) [31]. In the study, we assessed the organ function at 3-day intervals in accordance with the usual frequency of organ function assessment in the clinical practice. Daily assessment of organ function may provide more information compared with assessment at 3-day intervals. In addition, the relation between onset of MODS and EVLWI may be well elucidated by daily measurement of organ function and EVLWI. However, a new study is warranted to prove this point. Third, our study results, if further confirmed by large-scale prospective studies, provided a useful marker to stratify the patients. Therefore, measuring EVLWI in patients with severe sepsis should prompt efforts to identify and treat the modifiable factors associated with mortality. In addition, with the detection of increased EVLWI and subsequent survey and treatment of the possible causes of death in the early course of ICU stay, patients may have a better chance for survival.

In conclusion, EVLWI is an independent factor for the development of MODS in patients with severe sepsis. Elevated EVLWI levels on day 1 of severe sepsis may serve as a predictor for the development of MODS and mortality during the patients' ICU stay. Further more, the potential of novel treatment in severe sepsis with lung injury may develop. By current studies, someone can develop to design further researches to discover possible mechanism of sepsis with lung injury and develop novel treatment. However, further investigation is required to confirm these findings and to determine the utility of EVLWI as an early prognostic marker in patients with severe sepsis.

## Materials and Methods

### Patients

This study was conducted from April to December 2008 in a 37-bed medical intensive care unit (MICU). With approval of Institutional Review Board, Chang Gung Medical Foundation (IRB no.: 97-0374B) and written informed consent from all patients, we prospectively recruited 50 male and 17 female patients (totaling 67) on the day of MICU admission and within 24 hrs of the diagnosis of severe sepsis. All enrolled patients were recruited consecutively and met the criteria of sepsis with at least one organ failure. Patients were followed up until death or transferring out of ICU. Patients with the following criteria were excluded: pregnancy, age less than 18 years old, and uncontrolled malignancy. Severe sepsis was defined by the consensus committee of the American College of Chest Physicians and Society of Critical Care Medicine [23]. Patients' baselines included age, body mass index (BMI), vital signs, blood gas analysis, hematologic, and biochemical tests. Acute Physiology and Chronic Health Evaluation (APACHE) II scores and Sequential Organ Failure Assessment (SOFA) score were used for assessment of illness severity. The general care of the severe sepsis patients was according to the 2008 International guidelines of Surviving Sepsis Campaign [24]. In summary, blood, urine and other relevant specimens for culture were obtained before administration of antibiotics. Antibiotics were given according to clinicians' decisions related to the local prevalence of bacteria in the annual report of the Infection Control Committee of the institute. The patients were treated according International guidelines of Surviving Sepsis Campaign after the assessments.

### Definition

MODS was defined as more than 2 organs dysfunction (>3 organs) according to the Consensus Committee of American College of Chest Physicians and Society of Critical Care Medicine [10], [23]: Respiratory failure: Need for mechanical ventilation. Cardiovascular failure: Systolic BP ≤90 mmHg or mean arterial pressure ≤60 mmHg for 1 hour, despite adequate preload. Renal failure: Low urine output (eg, <0.5 mL/kg/hr), increased creatinine (≥50% increase from baseline) or need for acute dialysis. Hematological failure: Low platelet count (<100,000/mm3) or PT/PTT > upper limits of normal. Metabolic failure: Low pH with high lactate (eg, pH <7.30 and plasma lactate > upper limits of normal). Hepatic failure: Liver enzymes >2x upper limits of normal. CNS failure: Altered consciousness or reduced Glasgow Coma Score. Sequential Organ Failure Assessment (SOFA) score of these patients composed of scores from six organ systems, graded from 0 to 4 according to the degree of dysfunction/failure [25] had also to be calculated. The organ function was assessed at 3-day intervals or when there was clinical suspicion of a change in the patient's condition. Day 1 was defined as the day patients were recruited.

### Physiology, laboratory, oxygenation and ventilator parameters

The physiological parameters, including the previous 24-hour net input/output of fluid balance, and shock status were assessed on patient enrollment on day 1 and day 3. Shock was defined as systolic blood pressure <90 mm Hg or mean arterial pressure <60 mm Hg and vasopressor necessarily. The serum albumin level, white blood cell counts, platelet counts, arterial oxygen tension [PaO2]/fractional inspired oxygen [FiO2] ratio, lung injury score, and chest X-ray score were also collected on day 1 and day 3. Ventilator parameters collected were tidal volume indexed to predicted body weight, positive end expiratory pressure (PEEP), compliance of the lung, plateau pressure, mean airway pressure, and minute volume on day 1 and day 3.

### Measurement of extravascular lung water and hemodynamic parameters

The extravascular lung water (EVLW) measurement was based on the transpulmonary thermodilution method. The EVLW was measured everyday for 3 days but organ dysfunction was assessed on the day 1 and day 3 after ICU admission. The detail was well reported in our previous study [22]. This method only used a single indicator (cold saline solution), and demonstrated a satisfactory correlation with the gravimetric method [26]. A 4-F arterial catheter (PulsiocathPV2014L16; Pulsion Medical Systems, Munich, Germany) was positioned in the descending aorta via the femoral artery using the Seldinger technique. The femoral arterial catheter and a standard central venous catheter were connected to pressure transducers, and also to an integrated bedside monitor (PiCCO; Pulsion Medical Systems). Following three consecutive central venous injections of 10 ml iced 0.9% saline solution, continuous cardiac output (CO) calibration and EVLW measurements were obtained. The intrathoracic blood volume (ITBV) was obtained as our previous study [22]. Pulmonary permeability indexes (PPI) were calculated as EVLW/ITBV. CO calibrations and EVLW determinations were performed immediately following catheter insertion, and were employed as the hemodynamic parameters for managing patients in the medical ICU who had severe sepsis.

Parameters were indexed to total body surface area or to predicted body weight in order to facilitate comparisons. We selected 10 ml/kg as a cut-off value by ROC curve method analysis, which was highly sensitive (85%) and specific (75%) in predicting development of MODS. Therefore, elevated EVLI was defined as a value exceeding 10 ml/kg on the day of undergoing PiCCO system monitoring.

### Statistical analysis

All data are expressed as median (IQR (interquartile range)) or number (%). Since most continuous variables were skewed, nonparametric approaches were used in the study. Quantitative variables between two groups were compared using the Mann-Whitney test for continuous and ordinal variables and the chi-square test for nominal variables. The univariate analyses above were primarily used for the selection of variables, based on a *p* value less than 0.05. The selected variables were entered into a multinominal logistic regression analysis to identify the net effects of each individual factor. The potential problem of colinearity among several variables was evaluated using the Spearman correlation coefficient before running the analysis. Odds ratios (OR) and their 95% confidence intervals (CI) were computed by logistic regression model analysis to clarify the impact of several potentially independent prognostic factors. We plotted receiver operating characteristic (ROC) curves for day 1 EVLWI, SOFA score, APACHE II scores, lung injury scores, and prior 24 hrs fluid balance for prediction of development of MODS and mortality during ICU stay; the respective areas under the curves were calculated. The relationship between EVLWI and SOFA score was assessed with Spearman test. A p-value<0.05 was considered statistically significant. All analyses were conducted using SPSS software (version 10.0, SPSS, Chicago, IL) and Prism 4 for Windows (version 4.03, Graphpad Software Inc., San Diego, CA).

## References

[1] Martin GS, Mannino DM, Eaton S, Moss M (2003). The epidemiology of sepsis in the United States from 1979 through 2000.. N Engl J Med.

[2] Krau SD (2007). Making sense of multiple organ dysfunction syndrome.. Crit Care Nurs Clin North Am.

[3] Lydon A, Martyn JA (2003). Apoptosis in critical illness.. Int Anesthesiol Clin.

[4] Volman TJ, Hendriks T, Goris RJ (2005). Zymosan-induced generalized inflammation: experimental studies into mechanisms leading to multiple organ dysfunction syndrome.. Shock.

[5] Angus DC, Linde-Zwirble WT, Lidicker J, Clermont G, Carcillo J (2001). Epidemiology of severe sepsis in the United States: analysis of incidence, outcome, and associated costs of care.. Crit Care Med.

[6] Chen YC, Jenq CC, Tian YC, Chang MY, Lin CY (2009). Rifle classification for predicting in-hospital mortality in critically ill sepsis patients.. Shock.

[7] Zimmerman JE, Knaus WA, Wagner DP, Sun X, Hakim RB (1996). A comparison of risks and outcomes for patients with organ system failure: 1982-1990.. Crit Care Med.

[8] Marshall JC, Cook DJ, Christou NV, Bernard GR, Sprung CL (1995). Multiple organ dysfunction score: a reliable descriptor of a complex clinical outcome.. Crit Care Med.

[9] Vincent JL, Moreno R, Takala J, Willatts S, De Mendonca A (1996). The SOFA (Sepsis-related Organ Failure Assessment) score to describe organ dysfunction/failure. On behalf of the Working Group on Sepsis-Related Problems of the European Society of Intensive Care Medicine.. Intensive Care Med.

[10] Lin SM, Wang YM, Lin HC, Lee KY, Huang CD (2008). Serum thrombomodulin level relates to the clinical course of disseminated intravascular coagulation, multiorgan dysfunction syndrome, and mortality in patients with sepsis.. Crit Care Med.

[11] Aird WC (2003). The role of the endothelium in severe sepsis and multiple organ dysfunction syndrome.. Blood.

[12] Lewis RA, Austen KF, Soberman RJ (1990). Leukotrienes and other products of the 5-lipoxygenase pathway. Biochemistry and relation to pathobiology in human diseases.. N Engl J Med.

[13] Astiz ME, DeGent GE, Lin RY, Rackow EC (1995). Microvascular function and rheologic changes in hyperdynamic sepsis.. Crit Care Med.

[14] Molnar Z, Szakmany T, Heigl P (2003). Microalbuminuria does not reflect increased systemic capillary permeability in septic shock.. Intensive Care Med.

[15] Hotchkiss RS, Karl IE (2003). The pathophysiology and treatment of sepsis.. N Engl J Med.

[16] Abid O, Sun Q, Sugimoto K, Mercan D, Vincent JL (2001). Predictive value of microalbuminuria in medical ICU patients: results of a pilot study.. Chest.

[17] Spapen HD, Diltoer MW, Nguyen DN, Hendrickx I, Huyghens LP (2005). Effects of N-acetylcysteine on microalbuminuria and organ failure in acute severe sepsis: results of a pilot study.. Chest.

[18] Neumann P (1999). Extravascular lung water and intrathoracic blood volume: double versus single indicator dilution technique.. Intensive Care Med.

[19] Sakka SG, Ruhl CC, Pfeiffer UJ, Beale R, McLuckie A (2000). Assessment of cardiac preload and extravascular lung water by single transpulmonary thermodilution.. Intensive Care Med.

[20] Sakka SG, Klein M, Reinhart K, Meier-Hellmann A (2002). Prognostic value of extravascular lung water in critically ill patients.. Chest.

[21] Kuzkov VV, Kirov MY, Sovershaev MA, Kuklin VN, Suborov EV (2006). Extravascular lung water determined with single transpulmonary thermodilution correlates with the severity of sepsis-induced acute lung injury.. Crit Care Med.

[22] Chung FT, Lin SM, Lin SY, Lin HC (2008). Impact of extravascular lung water index on outcomes of severe sepsis patients in a medical intensive care unit.. Respir Med.

[23] Bone RC, Sibbald WJ, Sprung CL (1992). The ACCP-SCCM consensus conference on sepsis and organ failure.. Chest.

[24] Dellinger RP, Levy MM, Carlet JM, Bion J, Parker MM (2008). Surviving Sepsis Campaign: International guidelines for management of severe sepsis and septic shock: 2008.. Intensive Care Med.

[25] Vincent JL, de Mendonca A, Cantraine F, Moreno R, Takala J (1998). Use of the SOFA score to assess the incidence of organ dysfunction/failure in intensive care units: results of a multicenter, prospective study. Working group on “sepsis-related problems” of the European Society of Intensive Care Medicine.. Crit Care Med.

[26] Katzenelson R, Perel A, Berkenstadt H, Preisman S, Kogan S (2004). Accuracy of transpulmonary thermodilution versus gravimetric measurement of extravascular lung water.. Crit Care Med.

[27] Knaus WA, Draper EA, Wagner DP, Zimmerman JE (1985). APACHE II: a severity of disease classification system.. Crit Care Med.

[28] Martin GS, Eaton S, Mealer M, Moss M (2005). Extravascular lung water in patients with severe sepsis: a prospective cohort study.. Crit Care.

[29] Bognar Z, Foldi V, Rezman B, Bogar L, Csontos C (2010). Extravascular lung water index as a sign of developing sepsis in burns.. Burn in press.

[30] Gullo A, Berlot G, Viviani M (1996). The role of adult respiratory distress syndrome in the multiple organ dysfunction syndrome.. Acta Anaesthesiol Scand Suppl.

[31] Michard F (2007). Bedside assessment of extravascular lung water by dilution methods: temptations and pitfalls.. Crit Care Med.

